# Treatment Individualization in Colorectal Cancer

**DOI:** 10.1007/s11888-015-0288-z

**Published:** 2015-08-26

**Authors:** Robin M. J. M. van Geel, Jos H. Beijnen, René Bernards, Jan H.M. Schellens

**Affiliations:** Department of Clinical Pharmacology, The Netherlands Cancer Institute, Amsterdam, The Netherlands; Department of Pharmacy and Pharmacology, The Netherlands Cancer Institute, Amsterdam, The Netherlands; Division of Molecular Carcinogenesis, Cancer Genomics Center Netherlands, Amsterdam, The Netherlands; Department of Pharmaceutical Sciences, Utrecht University, Utrecht, The Netherlands

**Keywords:** Colorectal cancer, Treatment individualization, Biomarkers, MAPK, BRAF, KRAS, Combination therapy, Molecular subtypes

## Abstract

Colorectal cancer has been characterized as a genetically heterogeneous disease, with a large diversity in molecular pathogenesis resulting in differential responses to therapy. However, the currently available validated biomarkers KRAS, BRAF, and microsatellite instability do not sufficiently cover this extensive heterogeneity and are therefore not suitable to successfully guide personalized treatment. Recent studies have focused on novel targets and rationally designed combination strategies. Furthermore, a more comprehensive analysis of the underlying biology of the disease revealed distinct phenotypic differences within subgroups of patients harboring the same genetic driver mutation with both prognostic and predictive relevance. Accordingly, patient stratification based on molecular intrinsic subtypes rather than on single gene aberrations holds promise to improve the clinical outcome of patients with colorectal cancer.

## Introduction

**Fig. 1:**
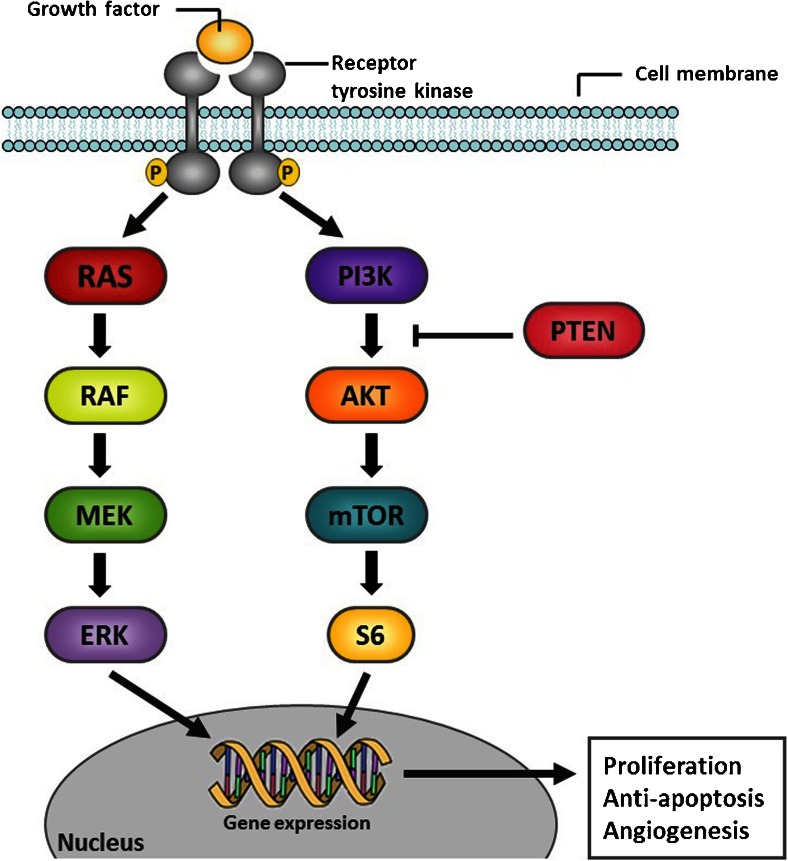
Simplified schematic overview of the MAPK and PI3K signaling pathway

Colorectal cancer (CRC) is one of the most prevalent cancers and a leading cause of cancer mortality worldwide [1]. In an effort to better understand the biologic hallmarks of the disease, CRC has undergone extensive molecular characterization in recent years, which revealed important oncogenes (e.g., *KRAS*, *BRAF*, *PIK3CA*), tumor suppressor genes (e.g., *APC*, *TP53*, *PTEN*) and signaling pathways that are critical for the development, survival, and progression of CRC cells. These genes are involved in major signaling pathways that have been linked to cancer, including the WNT/β-catenin, mitogen-activated protein kinase (MAPK), transforming growth factor beta (TGF-β) and phosphoinositide 3-kinase (PI3K) pathways (Fig. 1) [2]. Consequently, targeted agents against a number of druggable genomic aberrations were developed. However, patients who are characterized based on these molecular markers still show remarkable variability in terms of prognosis and response to therapy [3]. Therefore, many studies have addressed further subclassification of CRC, focusing on epigenetic factors and gene expression profiles. Herein we outline recent advances in our understanding of the underlying biology of CRC, and we address the potential clinical implications of this knowledge in order to optimize treatment individualization for patients with CRC.

## Single Genetic Aberration Driven Treatment

In the past decade, in search of better biomarkers in the metastatic setting of CRC (mCRC), a wide range of genetic alterations was found to be associated with cancer. Only a small percentage of these mutations actually drive the development and progression of malignant cells and may therefore have clinical implications [4, 5]. Mutations in the *RAS* genes (*KRAS*, *HRAS*, and *NRAS*), present in approximately 50 % of all patients with CRC, result in hyper activation of RAS proteins and their corresponding downstream pathways such as the MAPK pathway, thereby stimulating the development and progression of malignant tumors [6]. As *RAS* mutations predict resistance to anti-EGFR monoclonal antibodies (mAbs) cetuximab and panitumumab, patients with *RAS* mutant disease should be excluded from treatment with these drugs [7–11]. However, a large part of the *RAS* wild-type patients still do not respond to cetuximab or panitumumab. Further refinement in the molecular analysis of patients who are considered for treatment with anti-EGFR mAbs is therefore needed. Others, including Saridaki et al. in this issue, describe potential additional biomarkers and the uncertainties that remain to be solved. In short, mounting evidence in the literature supports the notion that resistance to anti-EGFR mAbs can be explained by genetic alterations in the MAPK and PI3K pathways [10, 12–14]. Although this evidence is largely based on retrospective analyses, which are often underpowered to address the impact of less common gene mutations (e.g., *NRAS*, non-codon 12 *KRAS*, *BRAF*, *PIK3CA*), there were trends of unresponsiveness upon cetuximab and panitumumab treatment. On the other hand, based on a meta-analysis of seven randomized controlled trials, Rowland et al. concluded that there is insufficient data to justify the exclusion of EGFR inhibitor therapy for patients with BRAFm mCRC [15]. Nevertheless, only 15 % of all patients with mCRC, who represent the quadruple wild-type (*KRAS*/*NRAS*/*BRAF*/*PIK3CA*) group, is thought to respond to anti-EGFR therapy [13, 16]. Therefore, effective alternatives for patients that do harbor mutations in these genes are needed.

## MAPK Pathway

Since the paradigm shift, changing from a histology-directed treatment approach to a genome-driven strategy, a large number of clinical trials investigated targeted agents against specific molecular anomalies. Patients were stratified according to their tumors’ *KRAS*, *BRAF*, *NRAS*, *PIK3CA*, or *PTEN* status to receive a matched targeted agent. However, none of these strategies has obtained proof of principle in patients with CRC [17–21]. Clearly, targeting a single mutated gene or activated protein in CRC does not yield the desired clinical benefit. In the meantime, preclinical research provided more insight into the dynamic interactions between different signaling pathways and emphasized that single kinase protein inhibition is way too simplistic as it ignores the complexity of feedback escape mechanisms and cross-talk between pathways. An impressive example is the contradictory efficacy of BRAF inhibitors in patients with melanoma versus patients with CRC. Selective BRAF inhibitors such as vemurafenib and dabrafenib have shown high objective response rates of about 50 % in patients with *BRAF*(*V600E*) mutated (BRAFm) melanoma [22, 23]. Combinations of these small molecules with MEK inhibitors like cobimetinib or trametinib further improved the rate of response to over 65 % and yielded a significant improvement in both progression-free survival and overall survival [24, 25]. In contrast, CRC patients with the identical causative *BRAF* mutation appeared unresponsive to BRAF inhibitors either as single agent or in combination with a MEK inhibitor as indicated by response rates of less than 5 % [20, 21]. Prahallad and colleagues elucidated the mechanism underlying this intrinsic unresponsiveness. Using an RNA interference-based genetic screen, they searched for kinase proteins, which upon suppression synergize with BRAF inhibition in BRAFm CRC cells. EGFR came out as the most potent synergy partner. Mechanistically, a feedback activation of EGFR was identified upon BRAF inhibition in BRAFm CRC supporting persistent tumor cell proliferation through reactivation of the MAPK and PI3K pathways. Combined BRAF and EGFR inhibition caused a strong synergistic effect in vitro and in xenograft models and resulted in complete inhibition of tumor growth [26••]. An independent research group reported similar results, BRAF inhibition in BRAFm CRC triggered an EGFR-mediated rebound activation of the MAPK pathway, which can be blocked by concomitant administration of an anti-EGFR targeted agent [27••]. This research supports clinical evaluation of combined BRAF and EGFR inhibition in patients with BRAFm CRC, and a number of ongoing clinical studies already have obtained clinical proof of principle. A phase I study investigating BRAF inhibitor encorafenib (LGX818) combined with cetuximab, either with or without PI3K inhibitor alpelisib (BYL719), demonstrated favorable toxicity profiles of both the dual and triple combination with objective response rates of 30 % and disease control rates of 80 and 90 %, respectively, in patients with pretreated advanced BRAFm CRC [28]. Comparable clinical activity was obtained in a second phase I study in which the combination of BRAF inhibitor dabrafenib, EGFR inhibitor panitumumab, and MEK inhibitor trametinib resulted in an objective response rate of 26 % [29]. Interestingly, vemurafenib plus cetuximab can also be safely combined with irinotecan and preliminary results of the first 16 patients with BRAFm mCRC revealed a 35 % response rate [30]. Given the particularly poor prognosis of patients with metastatic BRAFm CRC and the limited anti-tumor activity of standard treatment regimens in this subset of patients, new treatment options are needed [31]. Although larger phase II/III trials are necessary to confirm the promising initial results, combined BRAF and EGFR inhibition is likely to improve standard of care for BRAFm mCRC.

A second example of the dynamic biology of CRC concerns *KRAS* mutant (KRASm) disease. Despite extensive investigation, direct pharmacological inhibition of the KRAS protein remains difficult [32]. As an alternative, kinase proteins downstream of KRAS such as MEK were investigated but the anti-tumor activity was disappointing [33]. Upon the recognition that PI3K pathway activation due to coexisting genetic alterations or feedback upregulation may cause resistance against MEK inhibition, clinical studies focused on combining MEK inhibitors with PI3K, AKT or mTOR inhibitors, but these did not yield satisfactory results either [34]. Recently, investigators applied the previously described genetic screen to KRASm cells and found that HER3 knockdown sensitizes these cells for MEK inhibition. HER3 receptors are able to activate both the MAPK and PI3K pathway after dimerization with other members of the HER receptor family of which EGFR and HER2 are the most potent dimerization partners. Interestingly, combining a MEK inhibitor with an inhibitor of EGFR or HER2 did not result in synergy. Only MEK inhibition combined with dual EGFR/HER2 inhibitors or pan-HER inhibitors such as dacomitinib and afatinib, overcomes unresponsiveness of KRASm tumors in vitro as well as in vivo [35]. Based on a similar genetic drug screen, Corcoran et al. suggested another approach. They found that knockdown of the anti-apoptotic BH3 family gene BCL-XL was synergistic with MEK inhibition and targeting BCL-XL with ABT-263 (navitoclax) combined with a MEK inhibitor resulted in dramatic apoptosis in vitro and remarkable in vivo tumor responses in KRASm xenografts [36]. Clinical studies investigating these combinations are underway (NCT02039336, NCT02230553, NCT02450656, NCT02079740). Additionally, recent research provided new hope for the development of drugs that directly bind and inhibit the mutated RAS protein, by exploiting a mutation-specific approach [37, 38]. However, given the existing cross-talk between signaling pathways via feedback mechanisms, these novel therapeutic agents may urge for combination strategies as well. Furthermore, even if these investigational therapies provide benefit in the clinic, it seems likely, given the heterogeneity of CRC, that only a subset of KRASm and BRAFm patients will respond.

## PI3K Pathway

Similar to the MAPK pathway, the PI3K pathway can be activated by receptor tyrosine kinases, including EGFR family members, platelet-derived growth-factor receptors (PDGFR) and the insulin growth-factor-1 receptors. Mutations in *PIK3CA*, the gene that codes for the p110α isoform of the PI3K protein, are present in approximately 15 % of CRC and are concentrated in two hot spots of the gene at exon 9 (60–65 %) and exon 20 (20–25 %) [10, 39]. Exon 9 mutations affect the helical domain and cause gain of function independent of dimerization with the p85 subunit of PI3K, but require interaction with activated RAS. Exon 20 mutations on the other hand result in gain of PI3K function independent of activated RAS but highly rely on the interaction with p85 [40]. Therefore, these mutations might reflect two distinct subtypes of PI3K that behave differently upon a given treatment. Indeed, *PIK3CA* exon 9 mutations have been associated with activating *KRAS* mutations in codons 12 and 13, supporting the functional link between RAS signaling and activation of the helical domain of PI3K [10, 39, 41]. In contrast, others describe an association between *KRAS* mutations and *PIK3CA* mutations regardless of the exonic site [42]. These mechanistic differences may explain the discordant findings regarding the detrimental effect of *PIK3CA* mutations on the efficacy of anti-EGFR treatment. Initial reports showed an association between *PIK3CA* mutations and lack of response to anti-EGFR mAbs, but these results were not confirmed by others, possibly due to the lower number of exon 20 mutations in the latter studies [13, 43–45]. Inhibition of the PI3K pathway using small molecules targeting PI3K, mTOR, or AKT was recently investigated but has not yielded satisfactory results in CRC patients with PI3K alterations [46]. In addition to *PIK3CA* mutations, loss of PTEN expression is a second PI3K pathway component that has been associated with resistance to EGFR inhibition [47–50]. However, the effect of *PIK3CA* mutations and loss of PTEN on anti-EGFR therapy seems relatively weak and alterations in the PI3K pathway often co-occur with other important driver mutations like *KRAS* and *BRAF*, indicating that the PI3K pathway plays a less critical role compared to the MAPK pathway in unresponsiveness to EGFR inhibition in CRC and maybe even in CRC in general [13, 51].

Taken together, so-called quadruple-negative (no mutations in *KRAS*, *NRAS*, *BRAF*, *PIK3CA*) CRC is more likely to respond to EGFR inhibitors treatment and for those patients that do possess specific genetic mutations (*BRAF* and *KRAS*) clinical studies with new combination strategies are ongoing. However, presence of oncogenic mutations still do not fully explain the extent of non-response to EGFR inhibition and success stories with single gene mutation-directed therapy in patients with CRC are limited [52].

In contrast to CRC, targeting single mutated genes in hematological malignancies yielded a number of breakthrough therapies, such as BCR-ABL kinase inhibitor imatinib, and later dasatinib and nilotinib for patients with Philadelphia chromosome positive chronic myelogenous leukemia [53–55]. A number of reasons may explain this remarkable difference in success rate of targeting oncogenic drivers. First, unlike hematological malignancies, most solid tumors, and especially CRC, contain many genetic aberrations, which could make these tumors less dependent on a single oncogenic mutation. Moreover, this heterogeneity of CRC, in that it contains not only a mixture of relatively indolent and aggressive cells but also a mixture of molecularly different cell populations, makes it difficult to attack all these distinctive cells with the same targeted agents. Killing the sensitive cells might offer resistant cell populations the opportunity to expand and progress. Secondly, the affected signaling pathways often ‘communicate’ with each other, resulting in primary resistance against a given targeted agent as described previously for BRAFm and KRASm CRC. Thirdly, CRC behavior appears largely dependent on the surrounding stromal context in which the tumor exists [56, 57]. Therefore, treatment individualization for patients with CRC needs to take these features into account and move forward beyond the single gene paradigm.

## Towards Comprehensive Molecular Characterization Guided Treatment

Traditionally, early-stage CRC is classified based on tumor stage in order to estimate prognosis and predict benefit of adjuvant treatment. All patients with stage III or high-risk stage II disease are offered adjuvant chemotherapy. However, in a significant percentage of patients adjuvant chemotherapy is ineffective. Better prognostic and predictive markers are therefore needed to identify patients who are more likely to benefit from adjuvant treatment [58]. Initial studies described three molecularly distinct features in CRC, namely microsatellite instability (MSI), chromosomal instability (CIN), and the CpG island methylator phenotype (CIMP). CIN is present in the vast majority (~85 %) of CRCs and is characterized by alterations in the adenomatous polyposis coli (*APC*) gene, a key component of the WNT pathway. MSI on the other hand is detected in approximately 15 % of all early-stage CRCs and displays a hypermutable phenotype caused by a defective mismatch repair system (dMMR). In the clinical setting, MSI may be used to genetically diagnose Lynch syndrome, to estimate the prognosis of patients with CRC and to predict the efficacy of chemotherapeutic agent 5-fluorouracil (5-FU). Together with other studies, a large meta-analysis with over 7000 patients demonstrated that stage II or III colorectal tumors have a survival advantage if they exhibit MSI rather than a stable microsatellite status (MSS) as shown by the lower rates of tumor recurrence in MSI tumors [59]. The predictive role of MSI or dMMR for treatment with 5-FU and other chemotherapeutic agents has been controversial as several studies reported conflicting results. However, large recent retrospective analyses of randomized clinical trials confirmed that patients with dMMR should not be recommended for adjuvant treatment with 5-FU due to lack of benefit, but that this effect is limited to stage II patients [60–65]. In addition to MSI status as identified by PCR amplification of specific microsatellite repeats, Tian et al described a robust gene expression signature that identifies patients with MSI tumors and also a group of MSI-like patients who are not recognized by traditional methods but have a phenotype similar to MSI patients, including high mutation frequency, frequent *BRAF* mutations, high tumoral thymidylate synthase (TS) expression, and better prognosis [66]. As the unresponsiveness of MSI patients to adjuvant 5-FU therapy might be related to higher TS expression, MSI-like patients might therefore present an additional population that have a lower likelihood to benefit from 5-FU treatment [66, 67]. Furthermore, Choueiri et al. described a potential role of TS and excision repair cross-complementation group 1 (ERCC1) expression as prognostic and predictive biomarkers in mCRC. Low TS expression was associated with significant longer overall survival and combined low expression of ERCC1, and TS was predictive of response in patients treated with FOLFOX [68].

To gain more insight into the heterogeneity of CRC, the Cancer Genome Atlas Network (TCGA) conducted a comprehensive molecular characterization of CRC, including exome sequencing, DNA copy number, promoter methylation, and mRNA and miRNA expression analysis. Based on the mutational rate, an arbitrary cutoff was used to distinguish two separate groups, namely hypermutated (16 %) and non-hypermutated (84 %) tumor samples. The hypermutated group consisted of tumors with MSI and CpG island methylator phenotype (CIMP), whereas the non-hypermutated tumors had significantly more gene copy number alterations and *TP53* and *KRAS* mutations, indicating CIN. Analysis of the specific genes that were mutated revealed significant differences between the hypermutated and the non-hypermutated tumors, highlighting the marked differences in the biology and development of these CRC subtypes. *ACVR2A* (63 %), *APC* (51 %), *TGFBR2* (51 %), *BRAF* (40 %), *MSH3* (40 %), and *MSH6* were the most frequently mutated genes in the hypermutated tumors, whereas *APC* (81 %), *TP53* (60 %), *KRAS* (43 %), *TTN* (31 %), and *PIK3CA* (18 %) were the most frequent targets of mutations in non-hypermutated tumors. The RAS-MAPK, PI3K, and TGF-β signaling pathways were altered in 80 vs. 59 % (hypermutated vs. non-hypermutated), 53 vs. 50 %, and 87 vs. 27 %, respectively, but little is still known about which of these alterations are indeed necessary for continued disease progression. Despite these differences, 95 % of all tumors had a deregulated WNT signaling pathway, predominantly due to mutations in *APC*, and nearly all tumors had changes in MYC transcriptional targets, emphasizing the critical role of these features in CRC [69••]. Recent work demonstrated that *APC* inactivation is strictly required for CRC maintenance, even in the presence of additional CRC-associated oncogenic mutations in *KRAS* and *TP53*. In an shRNA-based transgenic mouse model, restoration of endogenous Apc protein induced a rapid and sustained tumor regression and re-established tissue homeostasis. These results suggest that small molecules that modulate the WNT pathway could be clinically active for patients with *APC*-mutated CRC [70]. Furthermore, Wiegering et al. described that inhibition of MYC mRNA translation using silvestrol, a compound that directly inhibits eIF4A in the translation complex of MYC, suppresses tumor growth in a mouse model of colorectal tumorigenesis [71]. Small molecules that modulate the WNT pathway or target MYC-translation initiation could therefore have clinical potential for patients with CRC.

Besides genetic changes, epigenetic alterations play a major role in the development of CRC. Hinoue and colleagues identified four DNA-methylation-based subgroups using a genome-scale DNA methylation profiling. A CIMP-high (CIMP-H) subgroup with an exceptionally high frequency of cancer-specific hypermethylation. A CIMP-low (CIMP-L) subgroup characterized by hypermethylation of a subset of CIMP-H associated markers. And two non-CIMP subgroups, one with a significantly higher frequency of *TP53* mutations and one with a marked low frequency of both cancer-specific DNA hypermethylation and gene mutations. Moreover, *KRAS* mutations occurred within each of these subgroups, indicating that not every *KRAS* mutant colorectal tumor has the same DNA methylation profile and that KRASm CRC is probably not a homogeneous group of patients who will respond similarly to therapy [72].

Moreover, Popovici et al. demonstrated that a subgroup of patients with *BRAF* wild-type stage II/III colon cancer have a similar phenotype as patients with *BRAF* mutant colon cancer, based on a high-sensitivity gene expression signature. Over 20 % of patients that were *BRAF* wild-type could be classified as being BRAFm-like according to this signature, showing clinicopathologic features similar to BRAFm patients, including higher frequencies of mucinous histology, MSI and poor overall survival and survival after relapse. As the majority of these BRAFm-like patients harbored *KRAS* mutations, this suggests a joint underlying biology between these KRASm tumors, but it also indicates histologic and prognostic heterogeneity within the KRASm subpopulation [73]. In addition, Tian and colleagues developed gene expression profiles that characterized KRAS-, BRAF-, and PIK3CA-activated stage II/III tumors, and they showed that tumors without mutations in either of these genes could have a similar gene expression profile [74]. This research indicates that mechanisms other than oncogenic mutations can cause a similar pathway activation that can be identified by a similar transcriptional pattern. More specifically, 79 of the 206 investigated tumors that had no oncogenic mutation in either *KRAS*, *BRAF*, or *PIK3CA* were classified as oncogenic based on their gene expression profiles. This indicates that a significant part of the *KRAS*, *BRAF*, and *PIK3CA* wild-type tumors share the same phenotype of an activated MAPK or PI3K pathway as those tumors with at least one activating mutation. Indeed, the combined oncogenic pathway signature was highly predictive for response to EGFR-inhibitor therapy with better performance than each of the three single mutation signatures and using *KRAS* mutation status alone [74]. Furthermore, as this signature detects patients whose tumors have an activated MAPK or PI3K pathway in the absence of genetic mutations, it may also be useful to select patients for treatment with small molecule inhibitors directed against non-mutated BRAF, MEK, PI3K, mTOR, or combinations of these agents [74]. However, paradoxical effects, such as seen with BRAF-inhibitors in non-BRAFm cells may be a concern of such strategy [75].

Subsequently, several research groups applied unsupervised clustering methods to genome-wide expression data in order to discover intrinsic molecular subtypes of CRC. De Sousa et al. for instance described three molecularly distinct CRC subtypes, the two well-known CIN and MSI tumors, called colon cancer subtype (CCS) 1 and 2, and a third subtype (CCS3) that is largely microsatellite-stable containing relatively more CpG-island methylator phenotype-positive tumors but cannot be identified on the basis of genetic mutations. *KRAS* mutations occurred in all the subgroups and *BRAF* mutations occurred in both CCS2 and CCS3, again indicating that *KRAS* and *BRAF* mutant CRC is not a homogeneous group and can be further differentiated into multiple biological groups with potential clinical differences in both prognosis and response to therapy. In fact, there was a marked difference in response between the three subtypes to cetuximab therapy in vitro, in xenografts, and in a clinical setting where patients with CCS3-classified tumors were resistant to cetuximab independently of *KRAS* mutation status [76•]. Others identified similar or slightly different subtypes which ranged in number from three to six, but there was a clear overlap in key clinical and molecular features between the subtypes from different research groups. Particularly, all groups concurred on the identification of a highly aggressive CRC subtype characterized by the expression of stem-cell genes, epithelial-to-mesenchymal transition (EMT), and poor prognosis [76•, 77•, 78•, 79•, 80•, 81•]. Therefore, the Colorectal Cancer Subtyping Consortium (CRCSC) was formed to reconcile data from six different research groups who subsequently established four consensus subtypes called CMS1-4. Although CMS1 was enriched for mutant *BRAF* status, *BRAF* mutations did also occur in CMS3, CMS4, and the unclassified subgroup. *KRAS* mutations were found in the largest proportion of CMS-3 tumors, but frequently in all other subtypes as well [82].

Taken together, all subtyping studies illustrate heterogeneity within the most common driver mutations, *KRAS* and *BRAF*, indicating important differences in prognosis and response to therapy with anti-EGFR agents and to a lesser extend to FOLFIRI treatment. Clearly, gene mutation status alone is not enough to define the complexity of the underlying biology of colorectal tumors. Therefore, to improve personalized treatment of CRC patients, a more comprehensive analysis is needed, which is less likely to be influenced by inter- and intra-tumor heterogeneity. CRC subtypes based on gene expression profiles may represent the initial step towards a better definition of CRC at the molecular level, but their prognostic and predictive value remains to be elucidated and prospectively validated.

## Clinical Implications

The major challenge now is to translate this emerging knowledge into a robust and reproducible classification system for CRC that integrates tumor biology, pathology, and clinical characteristics, and connect these subtypes to prognosis and response to therapy. Genome-wide expression data already provided initial evidence that particular drugs are more likely to be effective in specific colorectal cancer subtypes. As mentioned previously, tumors belonging to the CCS3 subtype as described by De Sousa et al., or the combined oncogenic activated pathway signature as described by Tian et al., are unlikely to respond to anti-EGFR directed treatment. In addition, Sadanandam and colleagues described a further subdivision of one CRC subtype representing patients with strong anti-EGFR response [80•]. Other favorable outcomes were reported in the metastatic setting for c-MET inhibitors in the cetuximab-resistant transit amplifying (CR-TA) subtype and for FOLFIRI in the stem-like subtype [80•].

One subgroup of CRC that was identified in each subtyping study was the mesenchymal phenotype, characterized by worst clinical outcome, resistance to adjuvant chemotherapy, and elevated transforming growth factor-β (TGF-β) signaling, a well-established feature in the induction of epithelial-to-mesenchymal transition (EMT). Inhibition of the TGF-β pathway may revert the mesenchymal tumors into a more epithelial-like phenotype, making these tumors more susceptible to chemotherapy [79•, 83]. This provides rationale to combine chemotherapy with TGF-β receptor inhibitors in patients with CRC harboring a mesenchymal phenotype as identified by the CMS4 subtype according to the CRCSC. In addition, Calon et al found that all mesenchymal poor prognosis subtypes identified by the above-described studies rely on genes expressed by stromal components, cancer-associated fibroblasts (CAFs), rather than by epithelial cancer cells. TGF-β signaling induces this CAF gene program, which boosts the metastatic potential and the ability to regenerate the malignant disease after therapy [56, 57, 84]. Pharmacological inhibition of TGF-β receptor 1 in the tumor microenvironment using TGF-βR1-specific inhibitor LY2157299 prevented metastasis formation and disease progression in patient-derived tumor organoids, which further warrants investigating TGF-β inhibition in patients with poor-prognosis CRC [57].

As previously mentioned, the genomic complexity of CRC limits the efficacy of targeted therapies. However, high mutational load may as well serve as a positive feature given the fact that somatic mutations found in tumors can be recognized by the patient’s own immune system due to their potential to encode non-self immunogenic antigens [85]. Nonetheless, in many tumors, the cytotoxic immune response is repressed by negative feedback systems, such as the programmed death 1 (PD-1) pathway. Inhibition of PD-1 or its ligand has demonstrated impressive clinical benefit in different types of cancer. Since colorectal cancers with deficient MMR are characterized by a hypermutated phenotype and contain lymphocyte infiltrates, it was hypothesized that especially dMMR tumors are responsive to inhibition of the PD-1 pathway [86, 87•]. A recently published phase II study indeed demonstrated that patients with dMMR CRC are much more responsive to anti-PD-1 immune checkpoint inhibitor pembrolizumab, than are MMR proficient CRC patients, indicated by objective response rates of 40 versus 0 %, respectively, and significantly prolonged progression-free survival and overall survival times [87•]. These impressive results strongly argue for clinical studies investigating anti-PD-1 antibodies in patients with MSI-like CRC as this gene expression classifier identifies an additional 10 % of CRC patients that harbor hypermutated tumors, adding up to an approximate 25 % of all patients with CRC who could potentially benefit from this novel treatment strategy.

## Conclusions and Future Perspectives

We begin to understand the heterogeneous character of CRC and its impact on prognosis and response to therapy. To optimize personalized therapy for patients with CRC, we should focus on new patient selection strategies based on this increased understanding of the underlying biology of the disease. In the metastatic setting, physicians should already expand their mutational analysis beyond *KRAS* (exon 2), as patients harboring other *KRAS* or *NRAS* mutations are unlikely to respond to EGFR inhibitors as well. The predictive role of BRAF mutations for response to anti-EGFR treatment remains inconclusive. Nevertheless, genomically driven clinical trials provide promising treatment strategies for patients who may not be eligible for anti-EGFR therapy (e.g., BRAFm and KRASm patients). Emerging molecularly defined CRC subtypes based on gene expression patterns highlight the heterogeneity beyond genetic mutations and the lack of unique driver mutations in each of these subtypes indicates distinct differences within the KRASm and BRAFm populations. Clearly, genetic aberrations are often not accurately defining a colorectal tumor’s phenotype and are highly insufficient to guide treatment decisions in most cases. In fact, thus far only one single-gene guided approach has obtained proof of principle in the clinic, namely combined BRAF and EGFR inhibition in BRAFm patients. Even in these successful initial studies, patients respond very differently. Therefore, validation of gene expression signatures, or equivalent simpler marker systems, and implementing these in the stratification of patients receiving pharmacological therapy may help defining the patient population most likely to benefit from a given (experimental) therapy. Furthermore, novel therapeutic strategies should be investigated beyond the currently used targets and drugs for CRC. Given the distinct differences between molecular CRC subtypes, vulnerability screens on representative cell lines for each subtype, rather than on cell line panels that contain multiple subtypes chosen on the basis of single genetic mutations may be useful to this purpose [88, 89]. Ultimately, this updated and improved treatment individualization based on comprehensive analysis of a patient’s tumor should enable physicians to make rational treatment decisions for each individual CRC patient.
